# Strengthening Migrant Access to Reproductive Healthcare in Thailand: observation of fixed and outreach service-based models of antenatal care for Karen and Myanmar migrants in Thailand, 2021–2024

**DOI:** 10.3389/fpubh.2026.1845308

**Published:** 2026-09-30

**Authors:** Rose McGready, May Mon Mon Theint, Jasper Ko Ko Aung, Nan San Wai, Prapatsorn Misa, Surirat Surina, Nay Win Tun, Aung Myat Min, Mary Ellen Gilder, Taco Jan Prins, Myo Chit Min, Kevin Jung-Yuan Lee, Waraporn Pimpasorn, Boonrid Wongchawengsup, Ahmar Hashmi, François H. Nosten

**Affiliations:** 1 Shoklo Malaria Research Unit, Mahidol-Oxford Tropical Medicine Research Unit, Faculty of Tropical Medicine, Mahidol University, Mae Ramat, Thailand; 2 Centre for Tropical Medicine and Global Health, Nuffield Department of Medicine, University of Oxford, Oxford, United Kingdom; 3 Department of Family Medicine, Faculty of Medicine, Chiang Mai University, Chiang Mai, Thailand; 4 Tak Public Health Office, Ministry of Public Health, Tak, Thailand; 5 Division of Public Health, Tak Provincial Administrative Organization, Ministry of Interior, Tak, Thailand; 6 Department of Nutrition Sciences and Health Behavior, School of Health Professions, University of Texas Medical Branch, Galveston, TX, United States

**Keywords:** access to healthcare, antenatal care, contraception, health insurance, midwifery, migrants, near-miss maternal mortality, outreach

## Abstract

**Background:**

Evidence to guide reproductive healthcare provision for undocumented migrants is limited. This study reports on the Strengthening Migrant Access to Reproductive Healthcare in Thailand (SMARH-T) initiative.

**Methods:**

This prospective observational study presents indicators of antenatal care (ANC), birth outcomes, and contraceptive uptake classified by the clinic of registration— standard (fixed clinic) or intervention (outreach clinic) —over a 3.5-year period from March 2021 to August 2024, including the initial 6-month pilot phase.

**Results:**

In total, 5,391 pregnant women (2,649 fixed and 2,742 outreach) registered over the 3.5 years, with the outreach team averaging 31 site visits per month. Seven of the nine WHO framework indicators for a positive pregnancy experience were reported for the two service-based models of antenatal care (ANC): the standard fixed clinic model and a new outreach model. Among women who delivered (2,280 fixed, 2,148 outreach), five of the seven indicators improved. The most improved measure by proportional increase from year one (baseline) to three, were eight ANC contacts (107.6% fixed, 81.7% outreach) and first ANC contact at <12 weeks (43.2% fixed, 31.0% outreach). First ANC contact at <12 weeks was the lowest-performing indicator overall (<30% of women). In contrast, syphilis screening, blood pressure monitoring, and fetal heartbeat measurement achieved >97.5% among all women. Homebirth without a skilled birth attendant was 2-fold higher in the outreach group (11.6% outreach, 5.3% fixed). Within 100 days of birth, 58.6% of women (1,945 of 3,318) opted for contraception; of these, the majority chose an implant (1,305 of 1,945, 67.1%), mostly (860 of 1,305, 65.9%) within 7 days postpartum. Completion of the 4-component delivery package (≥2 tetanus vaccinations, skilled birth attendance, health insurance and contraception within 100 days of birth) was achieved by 48.6% of women in total, with a greater proportional increase (year one to three) in the outreach group, including for health insurance. There were three maternal deaths (giving a maternal mortality ratio of 91 [95%CI: 31–267] per 100,000 live births [3 of 3,290]) and the maternal near-miss rate was 33 per 1,000 live births (95% CI: 28–40; 110 of 3,290), primarily due to postpartum hemorrhage.

**Conclusion:**

In total, five of seven quality indicators of care defined by the WHO framework for a positive pregnancy experience improved, with the outreach model of care nearly meeting the standards achieved by the fixed model. While the outreach model performed well, its sustainability remains a significant concern.

## Background

An estimated 3.6 million people from neighboring Myanmar, Cambodia, and Lao PDR live in Thailand (1). Tak Province in western Thailand is an agricultural region with a substantial population of Myanmar migrants. The majority of these migrants are in their reproductive years and require sexual and reproductive health (SRH) services, aligning with Sustainable Development Goal 3 (SDG3), which promotes “good health and wellbeing” and must include the most marginalized populations.

Registered migrants in Thailand support social security programs through taxes, which include health insurance (2). Undocumented migrants (also referred to as unauthorized, unregistered, or irregular immigrants) from Myanmar in Tak Province have primarily utilized SRH services from the non-governmental sector, such as clinics operated by non-governmental organizations, unless they need hospitalization for conditions requiring interventions such as an emergency cesarean section (3–6). This marginalized, non-Thai, undocumented border population has a high maternal mortality ratio of [176 per 100,000 live births (120 deaths among 68,024 live births)] (7) compared with that of Thai nationals (29 per 100,000 live births in 2024) (8).

The Shoklo Malaria Research Unit (7) is a non-governmental organization providing SRH services in Tak Province with two longstanding clinics that have served undocumented migrants since 1997 (9). A series of qualitative studies examining how to improve maternal healthcare access for undocumented pregnant women recommended the following measures: (i) expanding health insurance coverage to undocumented populations (10); (ii) ensuring birth documentation (11); and (iii) addressing travel barriers (12).

Although evidence supports community-based programming for SRH (13–16), the quality and breadth of ANC care delivered by outreach SRH services have been reported variably in the literature (17–19). Beginning in 2021, SMRU commenced a 6-month pilot of outreach SRH in four sites. The pilot included antenatal care (ANC), connecting to safe birth services, promotion and provision of contraceptives, and promotion of a low-cost health insurance scheme (M-FUND). Following the pilot, outreach services were expanded to a total of 15 sites (20). This project is known as the Strengthening Migrant Access to Reproductive Healthcare in Thailand (SMARH-T) initiative (21).

The aim of this analysis is to present observational cohort data comparing two concurrent service-based models of care: SMRU’s standard fixed-clinic model and a new outreach model implemented as part of the SMARH-T initiative, on positive pregnancy experience using seven of the nine WHO framework indicators (16, 22).

## Materials and methods

### Study design and participants

This observational cohort study was initiated prior to the COVID-19 pandemic. Two different aspects of the SMARH-T initiative have been recently published: (1) a mixed-methods evaluation demonstrating high service utilization, satisfaction, and reach among migrant women along the Thailand–Myanmar border (21); and (2) an evaluation of contraceptive utilization demonstrating that outreach clinics enhanced accessibility and significantly increased contraceptive uptake among marginalized populations (23).

This analysis was planned as an observational cohort study conducted at SMRU clinics in Tak Province, Thailand, using de-identified data to compare ANC and birth indicators over concurrent time periods according to the location where women first registered for pregnancy care: fixed or outreach clinics. Randomization of women to the two models of care was not ethical or feasible; rather, women self-selected between fixed or outreach clinics, largely influenced by access-related factors, including travel distance, cost, and security, as reported by Hashmi et al. 2026 (21). No women were excluded, and data were extracted from electronic records for pregnancies registered from 1 March 2021 to 31 August 2024.

### Setting

Two SMRU fixed clinics in Thailand, one in Wang Pha in Mae Ra Mat district and one in Maw Ker Thai in Phob Phra district, have been providing services, including safe childbirth, for more than 20 years to marginalized border populations. The outreach clinics were operated by and supported by these two fixed clinics. Outreach services started with a 6-month pilot phase from March 2021 to August 2021, before being expanded to 15 functional sites by the end of the survey period in August 2024 (Figure 1).

**Figure 1:**
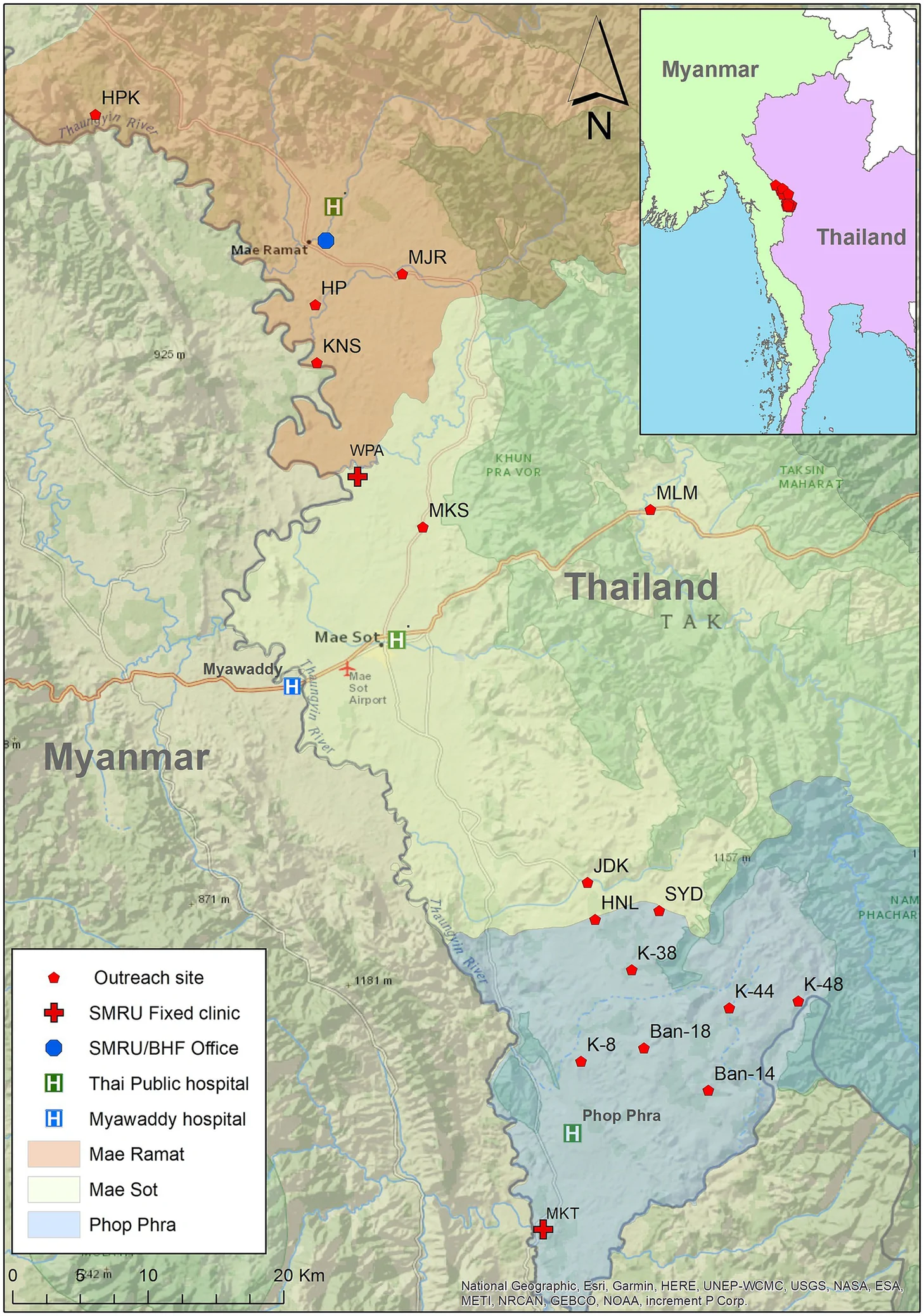
Map of fixed and outreach clinics in Mae Ra Mat and Phob Prah, rural Tak Province, Thailand by the end of the study period, 2024.

These locations were typically within a 60-min drive from the fixed clinic sites.

### Patient and public involvement

The SMARH-T initiative engaged the local population through the Tak Province Community Advisory Board in Thailand. Outreach clinics were only possible after an experienced community engagement team met and negotiated with local Thai Public Health authorities, village headmen, and migrant representatives in each village (24). Field visits examined potential areas before choosing a location convenient for migrants, protected from sun and rain, with adequate toilet facilities, and acceptable to both Thai and migrant residents of the surrounding villages.

### Services performed at outreach clinics

The fixed clinic health teams restructured their teams to support outreach while maintaining services at the fixed clinics. Care was delivered by locally trained staff. The ANC outreach team included one midwife, one sonographer, one counselor, one medic, one nurse, one information technology officer, and a physician (preferably Myanmar and/or Karen speaking). Community health volunteers, trained in SRH as the project was rolled out, were local and specific to the outreach site and suggested for the role by consensus as part of engagement activities. Evidence of staff background is provided elsewhere (25), but most staff remain without a recognized qualification from Myanmar or Thailand. A similar team was available at fixed clinics. Spoken and written languages included S’gaw and Pwo Karen, Burmese, Thai, and Mon (possible via a Mon-Burmese translator or a friend or relative who accompanied the woman).

The outreach initiative aimed to reach reproductive-age women and provide the same free services available at fixed clinics, including ANC, contraception, unwell child visits for children under 5 years of age, and Expanded Program on Immunization (EPI) vaccination. The significant exception was childbirth care, which was only available at the fixed clinics. The fixed clinics had inpatient care, e.g., treatment of pyelonephritis or malaria, and specific reproductive care such as internal and/or speculum examinations, e.g., to insert an intrauterine device (IUD). Women requiring care in labor, inpatient care, or requesting an IUD could be transported from the outreach to the fixed clinic. Women needing a cesarean section were referred from fixed clinics to Thai Public Hospitals (Figure 1). Fixed clinic ANC was available Monday to Friday, but outreach had to rely on a schedule of a particular day within the week and month. Busier outreach sites were visited weekly, while sites with fewer pregnant women were visited once per month, and most were visited twice per month, e.g., every 2nd Tuesday.

ANC contacts were scheduled for 12–18, 22–24, 25–28, 32, 36, 38, and 40 weeks, following a first ANC contact in the first trimester with a viable fetal heartbeat detected. Visits could be adjusted for late presenters or women with complicated pregnancies, e.g., hypertension. ANC services (outreach and fixed) included demographic details (age, gravidity, last menstrual period); physical examination (weight, height, blood pressure, pulse rate, respiratory rate, and physical examination); obstetric examination (fundal height, lie, and presenting part); and gestational age assessment by portable ultrasound devices. A mobile laboratory screened for vertical infections (HIV, syphilis, and hepatitis B), malaria by microscopy, and anemia by hematocrit at busy sites. For less busy sites, samples were brought back to the fixed clinic for testing, and results were available at the second ANC contact. Ultrasound was offered at first ANC contact and at 22–24 weeks routinely, and again if clinically indicated. Non-anemic women received ferrous sulfate 200 mg daily, folic acid 5 mg weekly, vitamin B1 100 mg daily. Anemic women received ferrous sulfate 400 mg daily, folic acid 5 mg, vitamin B12 200 mcg, vitamin C 100 mg, and thiamine hydrochloride 100 mg.

Every woman at the first ANC contact participated in a group counseling session for the purpose of blood tests, ultrasound exams, the risk of intimate partner violence (IPV) and depression, nutrition, and health insurance. This shares some similarities with group ANC (GANC), which is considered to improve satisfaction with ANC care (26, 27). Outreach and fixed clinics had the same system of group counseling for first ANC contact, delivered by a trained counselor in both service models. Before discharge from the first ANC consultation, each woman had a one-on-one counseling session to explain her point-of-care blood results and discuss IPV, mental health, and social support at home. Women with positive blood results were treated the same day if confirmation was not needed. All women were counseled about their plan for delivery with a skilled attendant, typically on more than one occasion. Regular health awareness sessions were conducted at all ANCs by community healthcare workers. For example, sessions discussed the dangers in pregnancy and for the neonate, health insurance, contraceptive options, hypertensive disorders, and other pertinent topics using large picture-based posters (28).

### Health insurance

For decades, mechanisms to support healthcare of undocumented migrants from Myanmar have fluctuated. Coverage, like the Thai Public Hospital-based insurance, now mandates that a Thai citizen, often the employer, confirm the applicant’s status, resulting in ineligibility for undocumented workers. The Migrant Fund (M-FUND) is a low-cost, not-for-profit health protection scheme for migrant workers in Thailand, created by Dreamlopments Social Enterprise and Foundation-FUND (20). While it was established to support sustainable access to quality healthcare services for migrants, it remains the only scheme open to migrants regardless of documentation status (29). Health insurance was tracked during this observational period as one possible method of confirming sustainability of basic services for marginalized populations, and is of rising importance with administrative changes in the USA (30).

### Variables

The number of undocumented migrants residing in the area is unknown due to seasonal and irregular work and civilian displacement, making it impossible to report indicators that require a known population denominator. The catalog of core indicators and metadata described in Lattof *et al*., for monitoring implementation of the new WHO ANC model for global comparison was applied in this marginalized border population (16). The nine core indicators included the proportion of pregnant women with: (1) the first ANC contact at estimated gestational age <12 weeks; (2) iron and folate provided for 90 + days; (3) screened for syphilis during ANC; (4) ANC contacts (a) at least 4, (b) at least 8; (5) blood pressure measurement (a) at least one blood pressure measurement during ANC, (b) at least one in 3rd trimester; (6) fetal heart beat listened to at least once during ANC; (7) women with an ultrasound scan before 24 weeks; (8) the proportion told about danger signs during ANC; and (9) experience of care, e.g., waiting time and support received during ANC. Indicators 1 to 7 are detailed here. Danger signs (8) are included in the ANC handheld patient record used at the border in picture format, and routinely taught by community healthcare workers during ANC; however, these sessions were not individually recorded, so data are not available. The ninth indicator, experience of care (9), has been published (21). Other ANC indicators included here are: preconceptual folate, testing for maternal infections (HIV, Hepatitis B, and malaria), anemia at first antenatal care contact, length of residence, and M-FUND uptake.

For the outcome of pregnancy, the following variables were included: the number of known (delivered: singleton or multiple; abortion) and unknown (lost to follow-up) outcomes. In women who delivered, a “complete” package included all four of the following: (i) at least two tetanus vaccinations; (ii) delivery with a skilled birth attendant, and (iii) health insurance; and (iv) contraception (implant, depot medroxyprogesterone acetate (depo), intrauterine device (IUD), and tubal ligation) by choice, within 100 days of birth.

Mortality indicators (early neonatal, maternal, and maternal near-miss mortality by the WHO Guidelines (31)) were compared for fixed and outreach models of antenatal care. The definitions of all variables were summarized (Supplementary Table S1).

### Study size

New ANC-registered women from the start of outreach, 1 March 2021 until 31 August 2024, contributed first ANC characteristics; and birth indicators were derived from women in the first 3 years of outreach (last registration 29 February 2024), as birth details take time to collate following registration. Women were grouped according to the site of their first ANC contact, but care was offered if they attended any outreach or fixed clinic. The database was not able to capture the site (outreach or fixed) for every ANC for every single woman.

### Statistical methods

Data were summarized as proportions and compared using the χ2 test with continuity correction or Fisher’s exact test. The χ2 test for linear trend was used to compare changes over time. Proportional change over time was calculated from year 1 to year 3 as (X_final_- X_initial_)/ X_initial_) *100 and expressed as a percentage. Mortality and maternal near-miss were described according to their definitions, expressed with 95% confidence intervals. Data were analyzed using SPSS V.27, and statistical significance was assessed at the 5% level.

## Results

Following the 6-month pilot phase, the outreach team averaged 31 site visits per month (Figure 1) from September 2021 to August 2024, including interruptions from COVID-19 lockdowns, Buddhist New Year, and floods (Figure 2). COVID-19 peak daily cases sourced from: https://www.worldometers.info/coronavirus/country/thailand/.

**Figure 2:**
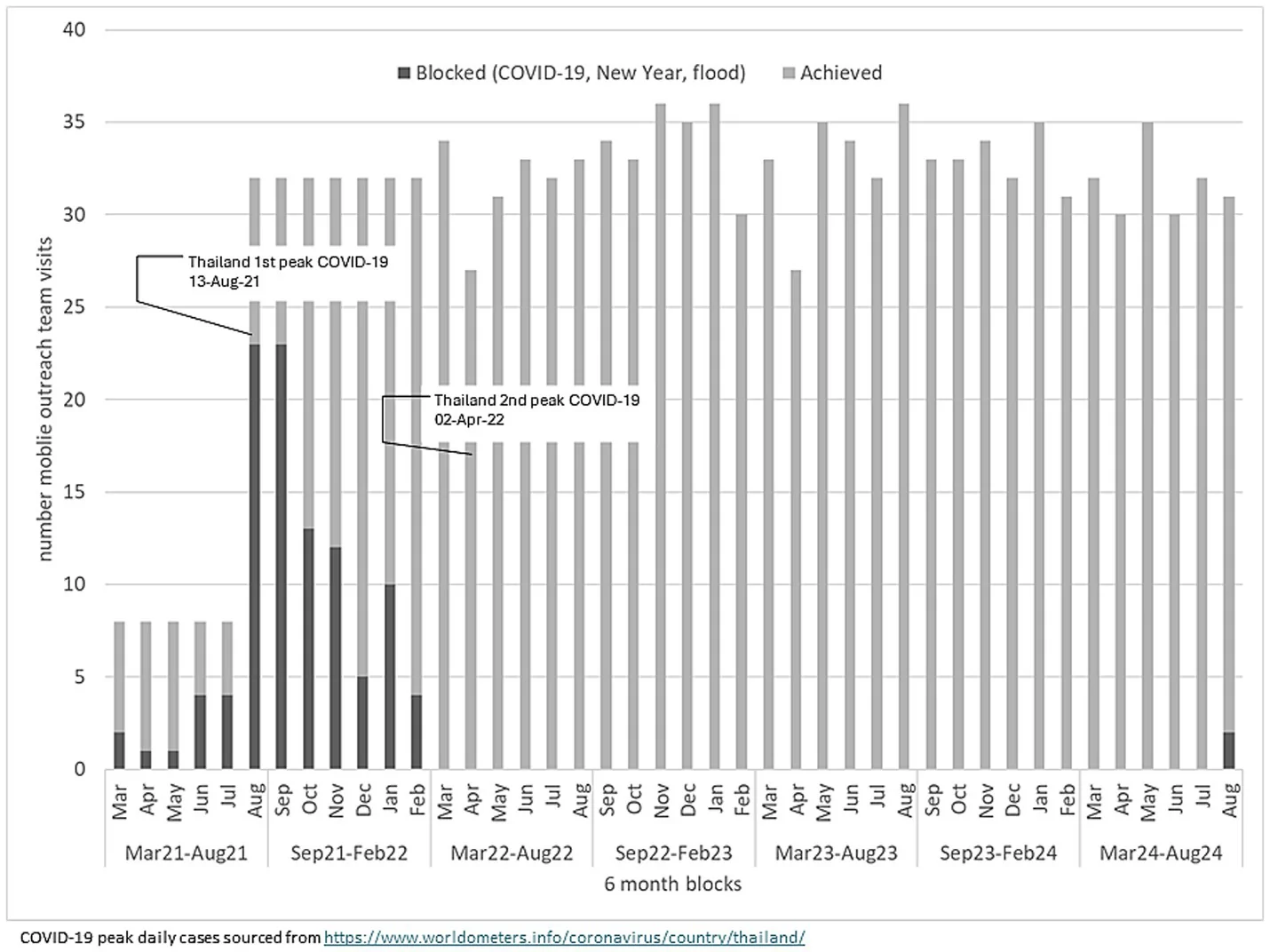
Outreach visits per month in Tak Province, Thailand, March 2021 to August 2024, showing “blocked” or outreach team visits unable to be completed vs. those “achieved” or completed.

There were 5,391 women registered for care: 2,649 at fixed clinics and 2,742 at outreach; and most women came from Phob Phra district (76.7%; 4,134 of 5,391). There was a yearly increase in the proportion of new registrations at outreach: 2021 29.7% (253 of 851), 2022 47.5% (756 of 1,590), 2023 57.2% (970 of 1,696), and 2024 60.8% (763 of 1,254). The proportion of short residence (≤6 months) at current address increased each year from 2021 to 2024 overall: 14.8% (136 of 851), 25.6% (407 of 1,589), 29.5% (500 of 1,696), and 29.7% (373 of 1,254), *p* < 0.001 linear trend; with similar results at fixed and outreach (data not presented).

### Baseline characteristics

The baseline characteristics of the women who registered at fixed and outreach clinics suggest the two populations were similar (Table 1). The number and proportion of preconceptual folate and malaria were very low (≤ 2.0%).

**Table 1:** Characteristics of women at first antenatal care consultation by clinic of registration, fixed or outreach, from 1 March 21 to 31 August 2024.

Newly registered pregnant women	Fixed	Outreach (OR)	Both models of ANC care	*p*-value
	*n* = 2,649	*n* = 2,742	*n* = 5,391	Fixed vs. Outreach
Teenager (Age <20y), % (*n*)	20.3 (537/2648)	19.4 (531/2742)	19.8 (1,068/5390)	0.419
Primigravida, % (*n*)	39.4 (1,043/2649)	39.0 (1,070/2742)	39.2 (2,113/5391)	0.814
Resident ≤6 months	26.0 (689/2648)	26.1 (717/2742)	26.1 (1,406/5391)	0.939
Preconceptual folate, % (*n*)	2.0 (54/2648)	1.0 (28/2472)	1.5 (82/5390)	0.003
HIV positive, % (*n*)	0.4 (11/2646)	0.4 (12/2472)	0.4 (23/5388)	1.000
Tuberculosis positive, % (*n*)	0.04 (1/2646)	0.07 (2/2742)	0.06 (3/5388)	1.000
Hepatitis B positive, % (*n*)	3.5 (92/2646)	3.7 (102/2742)	3.6 (194/5388)	0.685
Syphilis positive, % (*n*)	0.8 (21/2644)	0.8 (23/2742)	0.8 (44/5386)	0.976
Malaria positive, % (*n*)	0.8 (20/2645)	0.1 (3/2742)	0.4 (23/5387)	<0.001
Anemic, % (*n*)	6.3 (167/2638)	6.0 (165/2736)	6.2 (332/5374)	0.689

Missing data: teenage (1 fixed), preconceptual folate (1 fixed), resident (1 fixed), HIV/TB (3 fixed), syphilis (5 fixed), malaria (4 fixed), and anemia (11 fixed, 6 outreach).

Anemia defined as first trimester HCT <33%, and second and third trimester HCT<30%.

### Birth outcomes

Birth outcomes in fixed (*n* = 2,280) and outreach (*n* = 2,148) clinics were: unknown 14.1% (321 of 2,280) and 17.4% (372 of 2,280), abortion 10.4% (237 of 2,280) and 8.4% (180 of 2,280), singleton births 74.2% (1704 of 2,148) and 73.7% (1,583 of 2,148), or twin births 0.8% (18 of 2,148) and 0.6% (13 of 2,148), respectively. Most births took place at SMRU, followed by Thai hospital and home; for women receiving ANC at fixed clinics this was 79.5% (1,369 of 1,722), 15.2% (262 of 1,722), and 5.3% (91 of 1,722), compared to women receiving ANC on outreach this was 69.7% (1,112 of 1,596), 18.8% (299 of 1,596 and 11.6% (185 of 1,596), respectively. Overall, 10.8% (358 of 3,318) of births were by cesarean section (10.0% (173) and 11.6% (185) for fixed and outreach models of care.

### WHO seven core ANC indicators in women who gave birth

First ANC before 12 weeks and at least eight ANC contacts were the lowest-ranking core indicators, with syphilis screening, BP, and FHB measurements completed for >95% of women overall (Table 2). The majority of women (99.3%; 3,298 of 3,318) had an ultrasound at first ANC contact, with three in four before 24 weeks (Table 2). There was a positive linear trend for five of the seven core indicators, indicating increased quantity of care. Proportional change was highest for ANC-8 and first ANC < 12 weeks at both fixed and outreach models of care (Table 2).

**Table 2:** Linear trends and proportional change (year 1 to 3) in core indicators and birth package for fixed and outreach models of care (1 March 21 to 29 February 2024).

Year project	Fixed					Outreach					Proportional change Year 1 to 3	
	Mar-21 to Feb-22	Mar-22 to Feb-23	Mar-23 to Feb-24	Total fixed	*p-value	Mar-21 to Feb-22	Mar-22 to Feb-23	Mar-23 to Feb-24	Total Outreach	*p-value		
Outcomes	*n* = 731	*n* = 807	*n* = 742	*n* = 2,280		*n* = 347	*n* = 793	*n* = 1,008	*n* = 2,148		Fixed	Outreach
WHO framework: 7 core ANC indicators in women who gave birth												
Singleton/Twin births (*n*)	n-564	*n* = 622	*n* = 536	*n* = 1,722		*n* = 278	*n* = 607	*n* = 711	*n* = 1,596			
1. First ANC visit EGA < 12wks	23.6 (133)	28.0 (174)	33.8 (181)	28.3 (488)	<0.001	24.5 (68)	27.5 (167)	32.1 (228)	29.0 (463)	0.01	43.2	31.0
2. Received iron and folic acid for 90 + days	81.4 (459)	77.5 (482)	82.5 (442)	80.3 (1383)	0.677	78.4 (218)	83.5 (507)	85.2 (606)	83.4 (1331)	0.015	1.4	8.7
3. Screened for syphilis	100.0 (564)	99.8 (621)	99.8 (535)	99.9 (1720)	0.678	100.0 (278)	100.0 (607)	100.0 (711)	100.0 (1596)	0.351	−0.2	0.0
4a. At least 4 ANC contacts	84.0 (474)	87.3 (543)	90.7 (486)	87.3 (1503)	<0.001	74.8 (208)	90.1 (547)	92.7 (659)	88.6 (1414)	<0.001	8.0	23.9
4b. At least 8 ANC contacts	30.3 (171)	51.0 (31.7)	62.9 (337)	47.9 (825)	<0.001	34.9 (97)	55.2 (335)	63.4 (451)	55.3 (883)	<0.001	107.6	81.7
5a. ≥ 1BP measurement during ANC	93.6 (526)	99.8 (621)	100.0 (536)	97.8 (1683)	<0.001	93.9 (261)	100.0 (607)	100.0 (711)	98.9 (1596)	<0.001	6.8	6.5
5b. ≥ 1BP measurement in 3rd trimester	91.6 (515)	97.9 (609)	97.9 (525)	95.9 (1649)	<0.001	89.2 (248)	97.5 (592)	98.3 (699)	96.4 (1539)	<0.001	6.9	10.2
6. FHB listened to at least once	100.0 (564)	99.5 (619)	98.9 (530)	99.5 (1713)	0.010	100.0 (278)	100.0 (607)	100.0 (711)	100.0 (1596)	1.000	−1.1	0.0
7. US before 24 weeks	74.5 (420)	75.2 (468)	80.0 (429)	76.5 (1317)	0.031	73.0 (203)	80.4 (488)	81.2 (577)	79.4 (1268)	0.012	7.4	11.2

The proportion of women screened for syphilis did not improve but was very high from the outset in both fixed and outreach models of care (Table 2). There was no significant linear change at the fixed clinic in iron and folic acid supplementation for 90 days (already high at 80%), and at the outreach clinic in FHB at first ANC (also high due to routine ultrasound).

### Complete birth package

Just under half of the women achieved a complete package (four components), similar overall between models of care, and increasing over time. A greater proportional change was observed for the outreach model of care, mostly with improvements in skilled attendance at birth, contraceptive update by 100 days, and uptake of M-FUND (Table 3). The M-FUND peaked at outreach in the 3rd year. There was a notable decline in the proportion of women with no health insurance at both the fixed and outreach clinics.

**Table 3:** Birth package (4 components) at fixed and outreach clinics, 1 March 21 to 31 August 2024.

Year project		Fixed					Outreach					Proportional change Year 1 to 3	
		Mar-21 to Feb-22	Mar-22 to Feb-23	Mar-23 to Feb-24	Total fixed	^*^p-value	Mar-21 to Feb-22	Mar-22 to Feb-23	Mar-23 to Feb-24	Total Outreach	^*^p-value		
Singleton/Twin births (*n*)		*n*-564	*n* = 622	*n* = 536	*n* = 1,722		*n* = 278	*n* = 607	*n* = 711	*n* = 1,596		Fixed	Outreach
Complete birth package (4 components)		44.3 (250)	51.8 (322)	50.6 (271)	49.0 (843)	0.036	33.8 (94)	49.1 (298)	53.2 (378)	48.2 (770)	<0.001	14.2	57.4
(i) At least 2 tetanus vaccines		96.6 (545)	95.8 (596)	96.5 (517)	96.3 (1658)	0.868	95.0 (264)	97.9 (594)	98.2 (698)	97.5 (1556)	0.010	−0.1	3.4
(ii) Skilled attendant at birth		92.0 (519)	95.5 (594)	96.6 (518)	94.7 (1631)	<0.001	80.6 (224)	88.0 (534)	92.0 (654)	88.5 (1412)	<0.001	5.0	14.1
(iii) Contraceptive^ by choice (100 days)		56.9 (321)	57.9 (360)	58.4 (313)	57.7 (994)	0.618	52.9 (147)	59.5 (361)	62.3 (443)	59.6 (951)	0.009	2.6	17.8
	Chose Implant	72.6 (233)	68.1 (245)	72.2 (226)	70.8 (704)	0.62	68.7 (101)	65.7 (237)	59.4 (263)	63.2 (601)	0.020	−0.6	−13.5
(iv) Health Insurance	*M-Fund*	70.7 (399)	79.6 (495)	76.3 (409)	75.6 (1303)	0.005	60.1 (167)	81.7 (496)	85.1 (605)	79.4 (1268)	<0.001	7.9	41.6
	*Thai HI*	6.7 (38)	8.2 (51)	8.2 (44)	7.8 (133)		12.6 (35)	6.4 (39)	2.5 (18)	5.8 (92)		22.4	−80.2
	*No HI*	22.5 (127)	12.2 (76)	15.5 (83)	16.5 (286)		27.3 (76)	11.9 (72)	12.4 (86)	236 (14.8)		−31.1	−54.6

^Implant/depo/IUD/sterilization.

^*^Linear trend.

Italics indicate subgroup.

Postpartum contraception was used by 57.7 and 59.6% of women at fixed and outreach clinics, respectively (Table 3). The majority of these women chose implants (1,305 of 1,945; 67.1%), and most implant insertions (860 of 1,305; 65.9%) occurred within 7 days postpartum.

### Mortality indicators

The proportions (95% CIs) of stillbirth, early neonatal mortality, and maternal mortality, as well as the proportion of maternal near-miss cases, were similar between the two models of care (Table 4). There were few maternal deaths (3 of 3,290), resulting in a wide confidence interval for the maternal mortality ratio (91 per 100,000 live births). Causes of death included severe sepsis due to induced abortion in a multigravida (2021, fixed); homicide on day 3 postpartum due to a domestic violence attack that killed both mother and baby(2023, outreach); and hypovolemic shock due to postpartum hemorrhage despite skilled attendance at birth and referral to a Thai Hospital (2024, fixed). Apart from the induced abortion case, both women had at least ANC-4, M-FUND, and skilled attendance at birth.

**Table 4:** Mortality and near-miss indicators for fixed and outreach models of care.

Live births	Fixed	Outreach	Total
	*n* = 1,709	*n* = 1,589	*n* = 3,298
Stillbirth/1,000 total births	8 (CI 4–13) (*n* = 13)	4 (CI 2–8) (*n* = 6)	6 (CI 4–9) (*n* = 19)
Early neonatal mortality /1,000 live births	6 (CI 3–11) (*n* = 10)	3 (CI 1–7) (*n* = 5)	5 (CI 3–8) (*n* = 15)
Maternal mortality ratio /100,000 live births	117 (CI 32–426) (*n* = 2)	63 (CI 11–356) (*n* = 1)	91 (CI 31–267) (*n* = 3)
Maternal near-miss mortality /1,000 live births	30 (CI 23–39) (*n* = 51)	37 (CI 29–48) (n-59)	33 (CI 28–40) (*n* = 110)
Severe postpartum hemorrhage	20 (CI 14–28) (*n* = 34)	23 (CI 16–31) (*n* = 36)	21 (CI 17–27) (*n* = 70)
Severe pre-eclampsia	4 (CI 2–8) (*n* = 7)	6 (CI 3–11) (*n* = 9)	5 (CI 3–8) (n-16)
Eclampsia	2 (CI 1–6) (*n* = 4)	2 (CI 0.6–6) (*n* = 3)	2 (CI 1–4) (*n* = 7)
Sepsis	2 (CI 0.5–5) (*n* = 3)	4 (CI 2–8) (*n* = 6)	3 (CI 1–5) (*n* = 9)
Severe abortion complications	1 (CI 0.3–4) (*n* = 2)	3 (CI 1–7) (*n* = 5)	2 (CI 1–4) (*n* = 7)
Ectopic	3 (CI1-7) (*n* = 5)	2 (CI 0.6–6) (*n* = 3)	2 (CI 1–5) (*n* = 8)
Ruptured uterus	0	0	0
Other	0	1 (CI 0.1–4) (*n* = 1)	0.3 (CI 0.1–2) (*n* = 1)

Data expressed as ratio (CI 95%) (*n*).

Confidence intervals for maternal near-miss mortality were narrower, similar between models of care, and mostly a result of severe postpartum hemorrhage followed by severe pre-eclampsia and sepsis.

## Discussion

This study provides insight into a three year prospective observational cohort of undocumented migrant women along the Thailand–Myanmar border who elected to attend longstanding fixed clinics or a new outreach model of care. Of seven core ANC indicators, five improved during the SMARH-T project, with outreach clinics attaining similar proportions to fixed clinics, most likely by alleviating conditional access resulting from geographic, legal, and financial barriers (12, 32). Birth package indicators also improved, with the greatest proportional increase observed in the outreach model of care. It is pertinent that M-FUND uptake reached 75–79% by the third year from a baseline of 60–70%, accompanied by a fall in women without any health insurance to approximately 15%. These positive quantitative indicators complement the thematic analysis of the SMARH-T qualitative data, which demonstrated the acceptability, appropriateness, and improved reach with outreach services (21).

Caution is required in interpreting maternal mortality data given the wide confidence interval (MMR 91 (95% CI 31–267) per 100,000 live births, only 3 deaths). The MMR remains higher than the SDG target of 70 per 100,000 live births by 2030, and approximately four-fold higher than Thailand (27 in 2021 and 20 in 2020) (33) but lower than reported in Myanmar (179 in 2020) (34). Maternal near-miss case rates were similar between fixed and outreach clinics, important since only fixed clinics were able to provide birthing services. The top three causes of maternal near-miss mortality in Australia, New Zealand, South-East Asia and the South Pacific regions (35) were postpartum hemorrhage, pre-eclampsia and sepsis—as seen here. Care at the fixed clinics promoted positive outcomes for outreach-registered women. Birthing services are required 24 h per day and remain costly to run.

In this study, only 6 in 10 pregnant women completed at least 8 ANC contacts, short of the 100% target but markedly higher than the 1.6 in 10 recorded in a demographic health surveillance study from 19 low- and middle-income countries (36). While improving over time, the proportion of first ANC consultation before 12 weeks remained low. It will require community engagement initiatives and research examining perceptions toward early care, as recently reported in Fiji (37). Other indicators in the WHO framework remained consistently high, including syphilis screening, blood pressure measurement, fetal heart rate, and dating obstetric ultrasounds before 24 weeks.

In both the fixed and outreach models, contraceptive services are consistently offered, facilitating women’s access to modern contraceptive methods for birth spacing and potentially reducing mortality (38, 39). There were 57.7 and 59.6% of women at fixed and outreach clinics who opted for an effective method; and of these women, two in three chose implant. The significant uptake of free-of-charge contraceptives by Myanmar women at the border contrasts with data showing a low implant uptake of 0.3% from 2017–2018 in Yangon, which may be attributed to cost (40). In the Thai public health system, implant insertion costs $USD 74–83 (2,500–2,800 Thai baht), which can exceed the monthly earnings of agricultural migrant workers. Part of the success of the SMART-H initiative resulted from donor-supplied implants.

The challenges faced by host nations offering SRH services to migrants need to be acknowledged. For the Thailand–Myanmar border, challenges include: (i) the Thai health system has transitioned to an aging demographic in contrast to Myanmar, which still experiences high birth and fertility rates (41); (ii) healthcare professionals are typically not fluent in the languages spoken by migrants and migrants do not typically speak Thai (42); and (iii) undocumented migrant workers lack health insurance or pre-employment medical evaluations, leaving them at risk for catastrophic health costs if hospitalization is needed in Thailand (43). The care model discussed here was delivered by healthcare personnel from local communities who communicate in the languages of the women they serve and are trusted to advocate for initiatives such as screening blood for prevention of mother-to-child transmission of diseases, M-FUND, and implants. Support from Thai Public Health was essential: collaborating on the location of outreach sites, infectious disease surveillance and prevention (44), and for the 11% of women requiring life-saving cesarean sections (45). In the foreseeable future, strengthening collaboration with Thai Public Health and SMRU could provide a window for reducing the two-fold higher rate of homebirths in outreach-registered women. The outreach effectively highlighted M-FUND as a resource to help cover hospital expenses during childbirth if necessary, especially when offered through the outreach model of care (46). However, changing criteria for inclusion in insurance programs have shifted since this study was conducted that may result in a lower level of coverage than seen here (29). In May 2024, M-FUND’s recent iteration was revised to increase premiums and stipulate an earlier deadline for enrolment (<28 weeks). In the same timeframe, local Thai insurance schemes offered by district hospitals have also adopted more stringent criteria.

### Limitations of the study

The primary constraint of this research is its observational study design. Increased utilization of services with emergence from COVID-19 lockdowns was apparent but key indicator improvements continued after lockdowns were lifted (47, 48). Approximately one in seven were lost to follow-up before the outcome of pregnancy was known, marginally higher for outreach (17.4%) compared to fixed (14.1%) models of ANC care (Table 4). This aligns with, but is slightly lower than, the overall proportion of 18.6% documented over the past 39 years in this area (7). Lost to follow-up implies important data can go unreported, such as mortality. Another limitation is that women could change between fixed and outreach, and although this number was low, it was not officially recorded. While danger signs, one of the nine core indicators, were a routine part of services, we did not count each woman’s exposure to these education efforts, or her improvement pre- and post-health awareness sessions, and most importantly, retention of the knowledge. The previously published thematic analysis provides strong evidence of reduced transport time and costs at outreach as well as reduced risk of further payments at checkpoints (21).

The first ANC counseling at the woman’s first contact with care was in a group. This was the same as practiced at the fixed clinics and is mostly out of necessity due to limited staffing and is not modelled on Group-ANC, which is considered to improve satisfaction with ANC care (26, 27). Although limited, research on health system responsiveness (49) for migrants in Thailand is essential as poverty, conflict, and economic inequalities throughout Southeast Asia intensify human migration (50–53) and will continue to do so for the foreseeable future. The greatest issue is sustainability (30, 54). Further work on cost-effectiveness is underway.

## Conclusion

The outreach model of care achieved similar results to the standard fixed model of care for 7 of the 9 core indicators of the WHO Framework for a positive pregnancy experience measured in this study. This was complemented by positive proportional changes in the birth package, particularly for the outreach model of care. The outreach model of care was feasible and improved the quantity and quality of care for vulnerable migrants overall; however, sustainability under the current humanitarian funding landscape appears challenging.

## Data Availability

The raw data supporting the conclusions of this article will be made available by the authors, without undue reservation.
