# Supplementary material for: Strengthening Migrant Access to Reproductive Healthcare in Thailand: observation of fixed and outreach service-based models of antenatal care for Karen and Myanmar migrants in Thailand, 2021–2024
Source: Front Public Health. 2026 Sep 30;14:1845308. doi: 10.3389/fpubh.2026.1845308 (PMC13661194; doi:10.3389/fpubh.2026.1845308)
Supplement: Supplementary file 1 [file Data_Sheet_1.PDF]

Table S1. Indicators and definitions in analysis

**Baseline**	**Define**	**Variable**
District	District (or closest district) location of clinic	Mae Ramat/ Phob Phra
Year of date of first ANC consultation	Year	2021 to 2024
Teenager	Age <20 years old at registration to ANC	yes or no
Primigravida	Gravida 1, parity 0, i.e. her first pregnancy	yes or no
Length of residence ≤ 6 months	Lived at current address ≤6 months	yes or no
Preconceptual folate	Taking folate supplements before arriving to antenatal booking	yes or no
HIV	Screened at first ANC and tested positive for HIV	yes or no
Tuberculosis	Screened at first ANC and tested positive for TB	yes or no
Hepatitis B	Screened at first ANC and tested positive for Hepatitis B	yes or no
Syphilis	Screened at first ANC and tested positive for Syphilis	yes or no
Malaria	Screened at first ANC and tested positive for malaria by microscopy	yes or no
Anaemic	Haematocrit 1st ANC <33% (equivalent Hb <11g/dL) first trimester, <30% (equivalent Hb<10g/dL) second/third trimester	yes or no
**Births**		
Number of livebirths	Number of births	numeric
Place *SMRU*	At SMRU fixed clinic	yes or no
Home/on the way	At home or on the way to the clinic	yes or no
Hospital	At hospital	yes or no
Caesarean Section	Delivered by caesarean section in hospital setting	yes or no
**WHO framework 7 core ANC indicators (in women who gave birth)**		
1. First ANC visit EGA <12wks	Ultrasound confirmed first ANC attendance or pregnancy test if nothing visible on ultrasound	yes or no
2. Received iron and folic acid for 90+ days	Total days of supplements provided to the women at each visit	yes or no
3. Screened for syphilis	Screened at first ANC for Syphilis	yes or no
4a. At least 4 ANC contacts	4 or more ANC contacts	yes or no
4b. At least 8 ANC contacts	8 or more ANC contacts	yes or no
5a. ≥ 1BP measurement during ANC	BP measurement at least once	yes or no
5b. ≥ 1BP measurement in 3rd trimester	BP measurement in 3rd trimester	yes or no
6. FHB listened to at least once	FHB measured and recorded at least once	yes or no
7. US before 24 weeks	US Crown rump length/ Head circumference/abdominal circumference/femur length <24 weeks	yes or no

Continued….Table S1. Indicators and definitions in analysis

**Baseline**	**Baseline**	**Baseline**	**Baseline**
**Complete birth package**		Sum of 4 components listed below, Yes sum =4, To sum <4	yes or no
i) At least 2 tetanus vaccines		Tetanus status of at least 2 given before delivery	yes or no
ii) Skilled attendant at birth		Birth by trained attendant, excluding births at home or on the way	yes or no
iii) Contraception^ by choice within 100 days		Verified record of receiving contraception including the date received	yes or no
	Proportion choosing Implant	Record demonstrates choice of contraceptive was implant	yes or no
iv) Health Insurance	*M-Fund*	Type of health insurance by time of birth - the Migrant Fund	yes or no
	Thai HI	Thai hospital insurance (supported by village chief/employer)	yes or no
	No HI	No health insurance by time of birth	yes or no
**Mortality**			
Stillbirth		Proportion stillborn per 1,000 total births	yes or no
Early neonatal mortality		Proportion of liveborn neonates who died in first 7 days of life per 1,000 livebirths	yes or no
Maternal mortality ratio		Proportion of women who die in pregnancy and up 45 days post-partum per 100,000 live births	yes or no
Maternal near-miss mortality		Sum of any of the following per 1000 livebirths	yes or no
	Severe post-partum haemorrhage	Genital bleeding after delivery, with at least one of the following: perceived abnormal bleeding (1000 ml or more) or any bleeding with hypotension or blood transfusion.	yes or no
	Severe pre-eclampsia	Persistent systolic blood pressure of 160 mmHg or more or a diastolic blood pressure of 110 mmHg; urine protein (any degree) in place of proteinuria of 5 g or more in 24 hours; oliguria of <400 ml in 24 hours; and HELLP syndrome or pulmonary oedema. Excludes eclampsia.	yes or no
	Eclampsia	Generalized fits in a patient without previous history of epilepsy. Includes coma in pre-eclampsia.	yes or no
	Sepsis	Fever (body temperature >38°C), a confirmed or suspected infection (e.g. chorioamnionitis, septic abortion, endometritis, pneumonia), and at least one of the following: heart rate >100 (90), respiratory rate >24 (20), leukopenia (WBC <4000), leukocytosis (WBCs >12 000).	yes or no
	Severe abortion complications	Severe haemorrhage (=1000mL, hypotension, blood transfusion), severe sepsis or lesions to organs	yes or no
	Ectopic	Tubal pregnancy with pain or bleeding/imminent rupture	yes or no
	Ruptured uterus	Rupture of uterus in labour confirmed by laparotomy.	yes or no
	Other critical event or ICU	Critical events such as admission to CU	yes or no
